# Predicting clinically significant prostate cancer with a deep learning approach: a multicentre retrospective study

**DOI:** 10.1007/s00259-022-06036-9

**Published:** 2022-11-21

**Authors:** Litao Zhao, Jie Bao, Xiaomeng Qiao, Pengfei Jin, Yanting Ji, Zhenkai Li, Ji Zhang, Yueting Su, Libiao Ji, Junkang Shen, Yueyue Zhang, Lei Niu, Wanfang Xie, Chunhong Hu, Hailin Shen, Ximing Wang, Jiangang Liu, Jie Tian

**Affiliations:** 1 School of Engineering Medicine, Beihang University, Beijing, 100191 China; 2 Key Laboratory of Big Data-Based Precision Medicine (Beihang University), Ministry of Industry and Information Technology of China, Beijing, 100191 China; 3 School of Biological Science and Medical Engineering, Beihang University, Beijing, 100191 China; 4 Department of Radiology, The First Affiliated Hospital of Soochow University, Suzhou, 215006 Jiangsu China; 5 Department of Radiology, The Affiliated Zhangjiagang Hospital of Soochow University, Zhangjiagang, 215638 Jiangsu China; 6 Department of Radiology, Suzhou Kowloon Hospital, Shanghai Jiaotong University School of Medicine, Suzhou, 215028 Jiangsu China; 7 Department of Radiology, The People’s Hospital of Taizhou, Taizhou, 225399 Jiangsu China; 8 Department of Radiology, Changshu No.1 People’s Hospital, Changshu, 215501 Jiangsu China; 9 Department of Radiology, The Second Affiliated Hospital of Soochow University, Suzhou, 215004 Jiangsu China; 10 Department of Radiology, The People’s Hospital of Suqian, Suqian, 223812 Jiangsu China

**Keywords:** Magnetic resonance imaging, PI-RADS, Deep learning, Clinically significant prostate cancer

## Abstract

**Purpose:**

This study aimed to develop deep learning (DL) models based on multicentre biparametric magnetic resonance imaging (bpMRI) for the diagnosis of clinically significant prostate cancer (csPCa) and compare the performance of these models with that of the Prostate Imaging and Reporting and Data System (PI-RADS) assessment by expert radiologists based on multiparametric MRI (mpMRI).

**Methods:**

We included 1861 consecutive male patients who underwent radical prostatectomy or biopsy at seven hospitals with mpMRI. These patients were divided into the training (1216 patients in three hospitals) and external validation cohorts (645 patients in four hospitals). PI-RADS assessment was performed by expert radiologists. We developed DL models for the classification between benign and malignant lesions (DL-BM) and that between csPCa and non-csPCa (DL-CS). An integrated model combining PI-RADS and the DL-CS model, abbreviated as PIDL-CS, was developed. The performances of the DL models and PIDL-CS were compared with that of PI-RADS.

**Results:**

In each external validation cohort, the area under the receiver operating characteristic curve (AUC) values of the DL-BM and DL-CS models were not significantly different from that of PI-RADS (*P* > 0.05), whereas the AUC of PIDL-CS was superior to that of PI-RADS (*P* < 0.05), except for one external validation cohort (*P* > 0.05). The specificity of PIDL-CS for the detection of csPCa was much higher than that of PI-RADS (*P* < 0.05).

**Conclusion:**

Our proposed DL models can be a potential non-invasive auxiliary tool for predicting csPCa. Furthermore, PIDL-CS greatly increased the specificity of csPCa detection compared with PI-RADS assessment by expert radiologists, greatly reducing unnecessary biopsies and helping radiologists achieve a precise diagnosis of csPCa.

**Supplementary Information:**

The online version contains supplementary material available at 10.1007/s00259-022-06036-9.

## Introduction

Prostate cancer (PCa) is a malignancy of the urinary system in men, with the second highest incidence and the fifth highest mortality worldwide [1]. Multiparametric magnetic resonance imaging (mpMRI), which includes T2-weighted imaging (T2WI), diffusion-weighted imaging (DWI), apparent diffusion coefficient (ADC) maps derived from DWI, and dynamic contrast-enhanced (DCE) imaging, plays an important role in the risk stratification of PCa, particularly in reducing unnecessary biopsies and overtreatment for biopsy-naïve patients [2, 3]. Given the important role of mpMRI in the diagnosis of PCa, the Prostate Imaging and Reporting and Data System (PI-RADS) was developed, providing a standard guideline for mpMRI assessment of PCa, wherein the lesions are evaluated and interpreted based on a scoring system [2, 4, 5]. However, PI-RADS is a semi-quantitative assessment method that not only requires a high level of expertise from readers but also leads to inter-reader discrepancies, especially in multicentre clinical mpMRI assessment. More importantly, according to PI-RADS version 2.1 [2, 5], the detection of clinically significant prostate cancer (csPCa) is equivocal in the lesions with PI-RADS 3. Thus, lesions with PI-RADS 3–5 all require a biopsy for the pathological confirmation of csPCa in clinical practice [3, 6], leading to unnecessary biopsies and over-diagnosis or treatment.

Several recent studies have demonstrated that biparametric MRI (bpMRI), including T2WI, DWI, and ADC images, has a diagnostic performance similar to that of mpMRI in the detection of csPCa based on PI-RADS assessment [6–8]. As the absence of DCE in bpMRI can avoid some limitations of DCE, such as adverse effects, time consumption, and cost [6, 9, 10], bpMRI has been suggested as an alternative option for the diagnosis of csPCa [7].

As a typical artificial intelligence (AI) method, deep learning (DL) can characterise a large number of deep implied image features that are not available by visual assessment. This has great potential to reduce inconsistencies between observers and improve diagnostic accuracy [11–14]. Thus, DL has been widely used in the AI-aided diagnosis of various cancers, such as lung cancer, breast cancer, and other diseases [15, 16]. AI has been used to aid in the diagnosis and treatment of PCa [17–19]. Recently, some studies have developed DL models based on bpMRI images without DCE images to diagnose csPCa [13, 20]. These DL models showed similar classification performance to that of the PI-RADS assessment by expert radiologists based on bpMRI and DCE images. These findings implied that the DL models based on the bpMRI images without DCE might be comparable to the assessment by expert radiologists for the diagnosis of csPCa. However, for the comparison with PI-RADS assessment, these studies trained and tested classification models either using a single-centre cohort [13] or lacking validation with strict external and independent cohorts [20]. Therefore, the generalisation and reliability of the classification models in these studies need to be further validated using independent testing cohorts. Multicentre data are usually acquired using MRI scanners from different manufacturers with different acquisition parameters. The DL model developed according to multicentre data has relatively high reliability and generalisation [21, 22], thereby increasing the confidence of image readers in diagnosing csPCa. Thus, it is necessary to use multicentre training validation to examine further the performance of DL models for aiding the diagnosis of csPCa [21–23].

To bridge this gap, the present study aimed to develop DL models based on multicentre bpMRI (i.e. T2WI, DWI, and ADC derived from DWI) to aid the diagnosis of csPCa and then compare the performance of these models with that of the PI-RADS assessment by expert radiologists. We hypothesised that the ability of DL models based on bpMRI to diagnose csPCa is comparable to that of PI-RADS assessment by expert radiologists based on mpMRI.

## Material and methods

### Patients and ethical information

In this multicentre retrospective study, ethical approval was obtained from the ethics committee of the First Affiliated Hospital of Soochow University (SUH 1^st^), and the requirement for written informed consent was waived. The principles of the 1964 Helsinki Declaration and its later amendments were followed in this study.

**Fig. 1:**
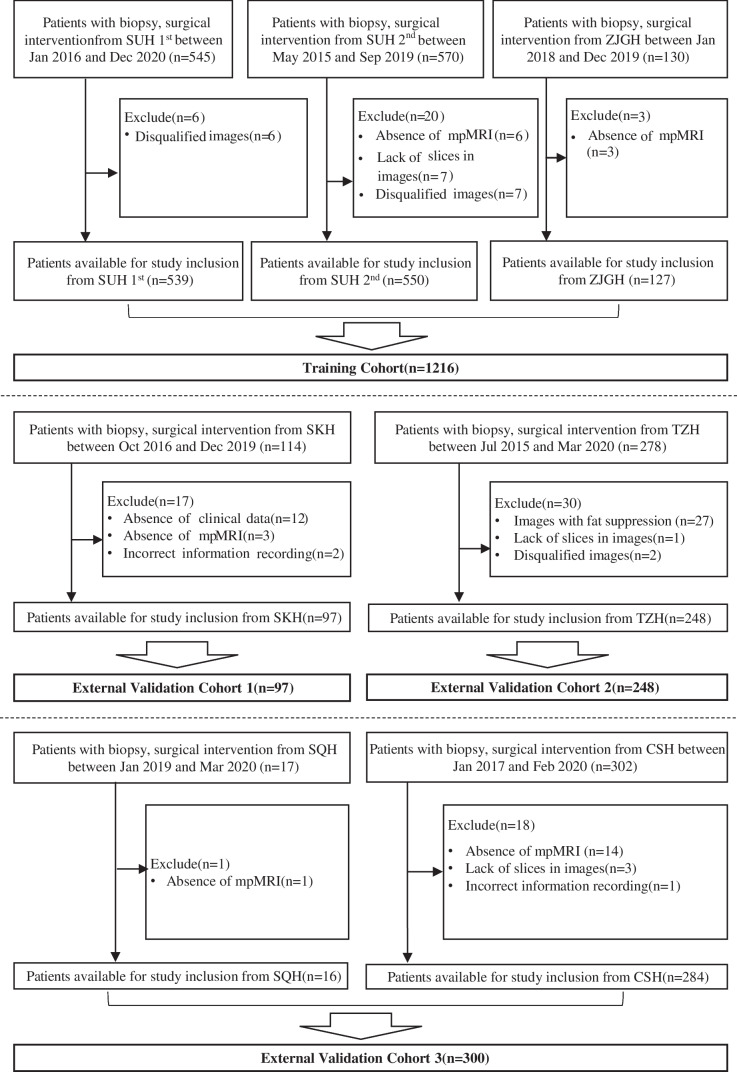
Flowchart for the inclusion and exclusion of patients from seven hospitals. Abbreviation: SUH 1^st^, the First Affiliated Hospital of Soochow University; SUH 2^nd^, the Second Affiliated Hospital of Soochow University; ZJGH, the Affiliated Zhangjiagang Hospital of Soochow University; SKH, Suzhou Kowloon Hospital; TZH, the People’s Hospital of Taizhou; SQH, the People’s Hospital of Suqian; CSH, Changshu No.1 People’s Hospital; mpMRI, multiparametric MRI

A total of 1956 consecutive male patients with histological confirmation between May 2015 and December 2020 at seven hospitals in different cities of Jiangsu province, China, were enrolled in this study, namely hospital 1, SUH 1^st^ with 545 patients; hospital 2, the Second Affiliated Hospital of Soochow University (SUH 2^nd^) with 570 patients; hospital 3, the Affiliated Zhangjiagang Hospital of Soochow University (ZJGH) with 130 patients; hospital 4, Suzhou Kowloon Hospital (SKH) with 114 patients; hospital 5, the People’s Hospital of Taizhou (TZH) with 278 patients; hospital 6, the People’s Hospital of Suqian (SQH) with 17 patients; and hospital 7, Changshu No.1 People’s Hospital (CSH) with 302 patients. According to the inclusion and exclusion criteria (Fig. 1), a total of 1861 consecutive patients from these hospitals were enrolled: 539 patients from SUH 1^st^, 550 patients from SUH 2^nd^, 127 patients from ZJGH, 97 patients from SKH, 248 patients from TZH, 16 patients from SQH, and 284 patients from CSH.

### MRI images acquisition

All MRI images were acquired using 3.0-T scanners (SUH 1^st^, Siemens Skyra; SUH 2^nd^, Philips Ingenia; ZJGH, Philips Achieva; SKH, Siemens Skyra; TZH, Siemens Skyra and Vero; SQH, Philips Ingenia; and CSH, Philips Achieva TX) with pelvic phased-array coils, details of which are described in Supplementary Sect. 1 (Table S1).

### PI-RADS assessment and histological review

According to PI-RADS version 2.1 [5], the mpMRI images (i.e. T2WI, DWI, ADC, and DCE) of all patients were retrospectively interpreted by five board-certified radiologists and two expert radiologists from SUH 1^st^ and SUH 2^nd^. The details of the PI-RADS assessment are described in Supplementary Sect. 2.

In the present study, according to clinical practice [3], patients with PI-RADS ≥ 3 lesions underwent targeted MR-guided biopsy (MRGB) and transrectal ultrasound (TRUS)-guided systemic biopsy, and those with PI-RADS 1–2 lesions underwent TRUS-guided systemic biopsy. Needle biopsies obtained by MRGB, TRUS-guided systemic biopsy, and prostatic specimens obtained by radical prostatectomy (RP) were independently reviewed by urological pathologists in the respective hospitals. The details of the histological review are described in Supplementary Sect. 3. For patients without RP, biopsy interpretation was used as the ground truth, and for those undergoing RP, postoperative pathological assessment was employed as the ground truth instead.

### MRI annotation

The entire volume of interest (VOI) of the lesion was manually delineated on T2WI using open-source ITK-SNAP software (http://www.itksnap.org/pmwiki/pmwiki.php, version 3.8.0) based on histopathological-imaging matching by the above-mentioned five board-certified radiologists. For each patient undergoing RP, we first manually assembled the histopathological specimens into pseudo-whole-mount sections according to the location marks of the prostate specimens. Urological pathologists marked the location of the lesions on the sections. In clinical practice, the boundaries of lesions in histopathological sections and those in MRI images are not the same. Thus, the lesion in the histopathological section was mapped to its counterpart in the spatially corresponding MRI slice as much as possible. Thus, the match between the lesion annotated via histological examination in the histopathological sections and that in the MRI images produced the ground truth map. The reference standard for the Gleason grade was based on RP findings. For patients who did not undergo RP, the reference standard for the Gleason grade group was based on biopsy findings using MRI/TRUS fusion-targeted biopsy followed by 12-gauge core systematic needle biopsy. A challenge in image labelling is the presence of ambiguous regions where the true tumour boundary cannot be deduced from MRI. To fill this gap, the VOI of each lesion was drawn twice by five board-certified independent radiologists. When patients had multiple lesions, only the index lesion with the highest Gleason score or the largest size (if the same Gleason score) was assessed. For each patient, the VOI delineated on the T2WI image was also used as a mask to extract corresponding lesions on the DWI and ADC images that had been aligned to the T2WI image.

### Data preprocessing

**Fig. 2:**
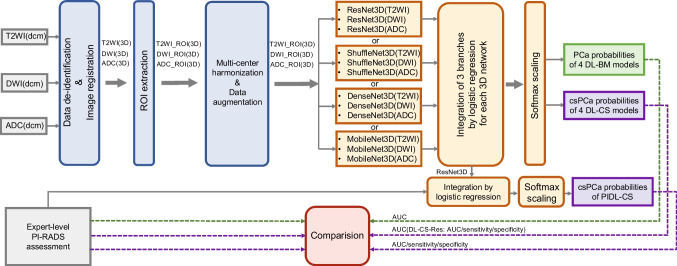
Flowchart of the development and comparison of the deep learning models and the integrated models with PI-RADS assessment. Abbreviation: T2WI, T2-weighted image; ADC, apparent diffusion coefficient; DWI, diffusion-weighted image; PCa, prostate cancer; csPCa, clinically significant prostate cancer; ROI, region of interest; PI-RADS, Prostate Imaging and Reporting and Data System; DL-BM, deep learning models for the classification between benign and malignant lesions; DL-CS, deep learning models for the classification between clinically significant and non-clinically significant PCa; DL-CS-Res, the deep learning model based on ResNet3D network for the classification between clinically significant and non-clinically significant PCa; PIDL-CS, integrated model combining DL-CS-Res and PI-RADS assessment; AUC, area under receiver operating characteristic curve

Image preprocessing was performed on T2WI, DWI images with high *b* value and ADC maps derived from DWI images, which included four steps, namely the data de-identification, the registration of the DWI images and ADC maps to the T2WI images, the multicentre data harmonisation, and the data augmentation (Fig. 2). Except the multicentre data harmonisation, the detail of other steps was described in Supplementary Sect. 4.

In the present retrospective study, multicentre data were acquired with different parameters and by the MRI scanners from different manufacturers, leading to inter-centre variation in the image spatial resolution and image intensity. The present study employed a data harmonisation method to reduce inter-centre image differences. Specifically, for each patient, the vertices of the lesion boundary in the slice with the largest profile of the lesion were identified, based on which a rectangle was drawn with an expansion of five pixels in this slice. Then, a three-dimensional region of interest (3D-ROI) was extracted by repeatedly copying this rectangle in the slices where the lesion was located and two additional 5-slice extensions to the top and bottom, respectively. This 3D-ROI contained intratumoural and peritumoural tissues. Peritumoural tissues include some components of the tumour microenvironment, such as tumour neovascularisation, lymphatic vessels, and tumour-related stroma or cells. Furthermore, all 3D-ROI images were interpolated into images using a cubic spline function with a common resolution that met the input requirements of the DL network. Finally, for each patient, each 3D-ROI image was *z*-score normalised by subtracting the means and then being divided by the standard deviations.

### DL model development

As shown in Fig. 2, in the present study, all DL models were developed using the preprocessed bpMRI (i.e. T2WI, DWI, and ADC derived from DWI) images. As shown in Fig. 1, for the above models, data from SUH 1^st^, SUH 2^nd^, and ZJGH were used as the training cohort, of which one-tenth of the patients were selected randomly and used as tuning dataset. Patients from SKH, TZH, CSH, and SQH were categorised as external validation cohorts. We integrated the data of CSH and SQH, with patients comprising one external validation cohort, because of the same manufacturer of the MRI scanner in these two hospitals and the small amount of SQH data (16 patients). The maximal area under the receiver operating characteristic curve (AUC) in the tuning dataset was used as the criterion for selecting the optimal hyperparameters and network weights. We divided the data into the training and external validation cohorts according to the following criteria. The data were divided at the hospital level rather than at the patient level. This can avoid the assignment of some patients of a hospital to the training cohort and the other data from the same hospital assigned to external validation cohorts, thereby guaranteeing that the DL models were tested using completely independent external cohorts. This allowed us to examine the performance of the DL models in each independent external validation cohort.

In the present study, DL models were developed based on a convolutional neural network (CNN), which is a typical and commonly used DL architecture used to aid the diagnosis of tumours [15, 16]. Four CNN-based models (ResNet3D, DenseNet3D, ShffleNet3D, and MobileNet3D) were used in this study (Fig. 2). These four three-dimensional (3D) CNNs are the 3D implementations of standard two-dimensional (2D) networks (i.e. ResNet18 [24], DenseNet [25], ShuffleNet [26], and MobileNet [27]), respectively. A 3D CNN can improve the performance of DL models by fully utilising spatial information ([13, 28]). In this study, as shown in Fig. 2, four types of DL models for the classification of benign and malignant lesions (DL-BM) were developed according to these four 3D CNNs, respectively. For each type of DL-BM model, the corresponding 3D CNNs (e.g. ResNet3D) were used to construct three branch networks respectively for T2WI images, DWI images, and ADC maps, which were integrated using a logistic regression model (Fig. 2). The details of the parameter settings for these networks are summarised in Supplementary Sect. 5. As mentioned previously, all lesions were pathologically confirmed, and the classification threshold was set according to the International Society of Urological Pathology Gleason grade group (GGG) [2]. Thus, malignant and benign lesions were defined as GGG ≥ 1 (Gleason score ≥ 3 + 3) and GGG < 1 (Gleason score < 3 + 3), respectively.

Then, as shown in Fig. 2, four DL models were developed based on the DL-BM models for the classification between csPCa and non-csPCa (DL-CS), respectively. Specifically, each DL-BM model was employed as the pre-trained structure for the corresponding DL-CS model. Then, the first layers of each pre-trained network were frozen, and the remaining layers were re-trained to construct the branch networks respectively for T2WI images, DWI images, and ADC maps, developing a new model of classification between csPCa and non-csPCa. A logistic regression model was used to integrate the three-branch network. According to the National Comprehensive Cancer Network (NCCN) guidelines, whether GGG is larger than and equal to 3 (Gleason score ≥ 4 + 3) was regarded as a pathological classification threshold for active surveillance [29]. A previous study showed a significant prognostic difference between GGG ≤ 2 (Gleason score ≤ 3 + 4) and GGG ≥ 3 (Gleason score ≥ 4 + 3) [30]. This threshold was also used for the detection of csPCa [31]. Thus, in the present study, csPCa and non-csPCa were defined as GGG ≥ 3 (Gleason score ≥ 4 + 3) and GGG ≤ 2 (Gleason score ≤ 3 + 4), respectively.

Finally, an integrated model (PIDL-CS) was developed by combining the DL-CS model with the best performance and the PI-RADS assessment. Specifically, among the DL-CS models, the one with the best performance in the tuning dataset was selected to generate the DL signature (Supplementary Sect. 6 [Table S2]). Subsequently, a logistic regression model combining the DL signature and PI-RADS score was developed for the detection of csPCa, which was referred to as the PIDL-CS model.

### Statistical analysis

1. ***Characteristic*** ***comparison*** ***between*** ***the training*** ***and*** ***external*** ***validation*** ***cohorts***
  In the present study, the Mann–Whitney *U* test was performed on continuous variables (e.g. age) to examine the difference between the training and external validation cohorts. The chi-square test was performed on categorical variables (e.g. prostate-specific antigen [PSA], tumour position, and PI-RADS score) to examine the differences between the training and external validation cohorts.
2. ***Model*** ***performance*** ***evaluation***
  To evaluate the performance of the proposed DL models, receiver operating characteristic (ROC) curves were depicted, and AUC, accuracy, sensitivity, and specificity were calculated for each external validation cohort (i.e. SKH, TZH, the combination of CSH and SQH [CSH + SQH]).
3. ***Comparison*** ***of diagnostic*** ***performances*** ***between*** ***DL*** ***models and PI-RADS*** ***assessment***
  The ROC curves of the DL models and PIDL-CS were compared with that of the PI-RADS assessment using the Delong test in each external validation cohort. Furthermore, the sensitivity and specificity of the DL-CS model with the best performance (evaluated by the tuning dataset, Supplementary Sect. 6 [Table S2]) and that of the PIDL-CS were compared with that of PI-RADS assessment using the McNemar test in each external validation cohort.

In the present study, the PI-RADS threshold for the detection of csPCa was ≥ 3. The risk probability thresholds for the detection of csPCa for the best DL-CS model and PIDL-CS were set comparable to that for PI-RADS assessment in the tuning dataset (Supplementary Sect. 7 [Table S3 and Fig. S1]). The same thresholds were used to evaluate the sensitivity and specificity of the best DL-CS model and PIDL-CS in each external validation cohort. Statistical analyses were performed using Python, RStudio (https://www.rstudio.com/, version 4.0.3), and Statistical Package for Social Sciences, version 26.0 (IBM, Armonk, NY, USA).

## Results

### Baseline characteristics

A total of 1861 consecutive patients with histological confirmation between May 2015 and December 2020 were enrolled in this study. Among them, 1216 patients from three hospitals were included in the training cohort: SUH 1^st^ (539 patients, median age: 72 years [66, 74]), SUH 2^nd^ (550 patients, median age: 69 years [64, 76]), and ZJGH (127 patients, median age: 73 years [68, 77]). In the training cohort, 122 patients were randomly selected for use as the tuning dataset. The patients of the remaining hospitals were employed as external validation cohorts, namely SKH (97 patients, median age: 69 years [64, 75]), TZH (248 patients, median age: 73 years [68, 79]), and CSH + SQH (300 patients, median age: 70 years [65, 75]). Some of these patients underwent radical prostatectomies at each hospital (SUH 1^st^, 259 patients; SUH 2^nd^, 112 patients; ZJGH, 20 patients; SKH, 54 patients; TZH, 28 patients; and CSH + SQH, 10 patients).

The demographic and clinical characteristics of the patients are summarised in Table 1. Age, Gleason grade, PSA level, tumour location, and PI-RADS scores were significantly different between the training and external validation cohorts (*P* < 0.05). In contrast, no significant difference in the proportion of csPCa or that of malignancy was observed between the training and external validation cohorts (*P* > 0.05).

**Table 1:** Demographic and clinical characteristics of patients

Variable	Training Cohort				External Validation Cohorts				*P* value
	SUH 1^st^ (*n* = 539)	SUH 2^nd^ (*n* = 550)	ZJGH (*n* = 127)	ALL (*n* = 1216)	SKH (*n* = 97)	TZH (*n* = 248)	CSH + SQH (*n* = 300)	ALL (*n* = 645)	
MRI strength and vendor	3 T, Siemens	3 T, Philips	3 T, Philips		3 T, Siemens	3 T, Siemens	3 T, Philips	NA	NA
Median age (y)†	72 (66, 74)	69 (64, 76)	73 (68, 77)	70 (64, 76)	69 (64, 75)	73 (68, 79)	70 (65,75)	71 (66,76)	0.001*
Biopsy Gleason grade group	**537**	**550**	**127**	**1214**	**81**	**246**	**288**	**615**	< 0.001*
0	179 (33.3%)	374 (68.0%)	47 (37.0%)	600 (49.4%)	44 (54.3%)	103 (41.9%)	172 (59.5%)	319 (51.9%)	
1	36 (6.7%)	66 (12.0%)	17 (13.4%)	119 (9.8%)	9 (11.1%)	8 (3.3%)	9 (3.1%)	26 (4.2%)	
2	81 (15.1%)	27 (4.9%)	17 (13.4%)	125 (10.3%)	12 (14.8%)	16 (6.5%)	39 (13.5%)	67 (10.9%)	
3	84 (15.6%)	26 (4.7%)	10 (7.9%)	120 (9.9%)	2 (2.5%)	30 (12.2%)	17 (5.9%)	49 (8.0%)	
4	68 (12.7%)	22 (4.0%)	16 (12.6%)	106 (8.7%)	9 (11.1%)	46 (18.7%)	34 (11.8%)	89 (14.5%)	
5	89 (16.6%)	35 (6.4%)	20 (15.7%)	144 (11.9%)	5 (6.2%)	43 (17.5%)	17 (5.9%)	65 (10.6%)	
Surgical Gleason grade group	**259**	**112**	**20**	**391**	**54**	**28**	**10**	**92**	< 0.001*
0	0 (0.0%)	0 (0.0%)	0 (0.0%)	0 (0.0%)	26 (48.1%)	2 (7.1%)	1 (10.0%)	29 (31.5%)	
1	18 (6.9%)	45 (40.2%)	5 (25.0%)	68 (17.4%)	3 (5.6%)	0 (0.0%)	1 (10.0%)	4 (4.3%)	
2	61 (23.6%)	22 (19.6%)	5 (25.0%)	88 (22.5%)	10 (18.5%)	6 (21.4%)	3 (30.0%)	19 (20.7%)	
3	79 (30.5%)	19 (17.0%)	2 (10.0%)	100 (25.6%)	2 (3.7%)	3 (10.7%)	2 (20.0%)	7 (7.6%)	
4	30 (11.6%)	12 (10.7%)	2 (10.0%)	44 (11.3%)	7 (13.0%)	6 (21.4%)	1 (10.0%)	14 (15.2%)	
5	71 (27.4%)	14 (12.5%)	6 (30.0%)	91 (23.3%)	6 (11.1%)	11 (39.3%)	2 (20.0%)	19 (20.7%)	
Overall Gleason grade group	**539**	**550**	**127**	**1216**	**97**	**248**	**295**	**640**	< 0.001*
0	179 (33.2%)	374 (68%)	47 (37.0%)	600 (49.3%)	49 (50.5%)	104 (41.9%)	172 (58.3%)	325 (50.8%)	
1	20 (3.7%)	66 (12%)	14 (11.0%)	100 (8.2%)	7 (7.2%)	6 (2.4%)	9 (16.6%)	22 (3.4%)	
2	74 (13.7%)	27 (4.9%)	17 (13.4%)	118 (9.7%)	18 (18.6%)	18 (7.3%)	42 (14.2%)	78 (12.2%)	
3	97 (18.0%)	26 (4.7%)	12 (9.4%)	135 (11.1%)	3 (3.1%)	30 (12.1%)	19 (6.4%)	52 (8.1%)	
4	58 (10.8%)	22 (4%)	14 (11.0%)	94 (7.7%)	11 (11.3%)	41 (16.5%)	35 (11.9%)	87 (13.6%)	
5	111 (20.6%)	35 (6.4%)	23 (18.1%)	169 (13.9%)	9 (9.3%)	49 (19.8%)	18 (6.1%)	76 (11.9%)	
Label 1	**539**	**550**	**127**	**1216**	**97**	**248**	**300**	**645**	0.62
Benign	179 (33.2%)	374 (68%)	47 (37.0%)	600 (49.3%)	49 (50.5%)	104 (41.9%)	173 (57.7%)	326 (50.5%)	
Malignant	360 (66.8%)	176 (32%)	80 (63.0%)	616 (50.7%)	48 (49.5%)	144 (58.1%)	127 (42.3%)	319 (49.5%)	
Label 2	**539**	**550**	**127**	**1216**	**97**	**248**	**295** #	**640**	0.32
non-csPCa	273 (50.6%)	440 (80%)	78 (61.4%)	791 (65.0%)	74 (76.3%)	128 (51.6%)	229 (77.6%)	431 (67.3%)	
csPCa	266 (49.4%)	110 (20%)	49 (38.6%)	425 (35.0%)	23 (23.7%)	120 (48.4%)	66 (22.4%)	209 (32.7%)	
PSA (ng/ml)	**539**	**550**	**127**	**1216**	**97**	**248**	**300**	**645**	< 0.001*
0 < = PSA < 10	197 (36.5%)	268 (48.7%)	26 (20.5%)	491 (40.4%)	39 (40.2%)	99 (39.9%)	115 (38.3%)	253 (39.2%)	
10 < = PSA < 20	152 (28.2%)	189 (34.4%)	41 (32.3%)	382 (31.4%)	14 (14.4%)	30 (12.1%)	88 (29.3%)	132 (20.5%)	
PSA ≥ 20	190 (35.3%)	93 (16.9%)	60 (47.2%)	343 (28.2%)	44 (45.4%)	119 (48.0%)	97 (32.3%)	260 (40.3%)	
Position	**539**	**550**	**127**	**1216**	**97**	**248**	**300**	**645**	< 0.001*
TZ	186 (34.5%)	311 (56.5%)	58 (45.7%)	555 (45.6%)	63 (64.9%)	122 (49.2%)	186 (62%)	371 (57.5%)	
PZ	259 (48.1%)	146 (26.6%)	45 (35.4%)	450 (37.0%)	34 (35.1%)	37 (14.9%)	77 (25.7%)	148 (22.9%)	
TZ and PZ	94 (17.4%)	93 (16.9%)	24 (18.9%)	211 (17.4%)	0 (0)	89 (35.9%)	37 (12.3%)	126 (19.5%)	
PI-RADS scores	**539**	**550**	**127**	**1216**	**97**	**248**	**300**	**645**	< 0.001*
1	1 (0.19%)	14 (2.5%)	0 (0)	15 (1.2%)	0 (0)	5 (2.02%)	1 (0.3%)	6 (0.9%)	
2	129 (23.9%)	246 (44.7%)	31 (24.4%)	406 (33.4%)	23 (23.7%)	63 (25.4%)	97 (32.3%)	183 (28.4%)	
3	72 (13.4%)	129 (23.5%)	27 (21.3%)	228 (18.8%)	23 (23.7%)	64 (25.8%)	101 (33.7%)	188 (29.1%)	
4	92 (17.1%)	99 (18%)	14 (11.0%)	205 (16.9%)	25 (25.8%)	25 (10.9%)	24 (8%)	74 (11.5%)	
5	245 (45.5%)	62 (11.3%)	55 (43.3%)	262 (21.5%)	26 (26.8%)	91 (36.7%)	77 (25.7%)	194 (30.1%)	

The bold values indicate the total number for the corresponding variable

#There were actually 295 patients of CSH + SQH hospital in the classification between csPCa and non-csPCa, due to 5 patients missing further immunohistochemical examination and making it difficult to accurately assess specific Gleason grade group

^*^Significant (*P* < 0.05)

^†^Data in parentheses are the interquartile range

Abbreviation: *ISUP* International Society of Urological Pathology, *PSA* prostate-specific antigen, *PI-RADS* Prostate Imaging and Reporting and Data System, *PZ* peripheral zone, *TZ* transition zone, *csPCa* clinically significant prostate cancer, *non-csPCa* non-clinically significant prostate cancer, *SUH 1*^*st*^ the First Affiliated Hospital of Soochow University, *SUH 2*^*nd*^ the Second Affiliated Hospital of Soochow University, *ZJGH* the Affiliated Zhangjiagang Hospital of Soochow University, *SKH* Suzhou Kowloon Hospital, *TZH* the People’s Hospital of Taizhou, *SQH* the People’s Hospital of Suqian, *CSH* Changshu No.1 People’s Hospital

Because there was a significant difference in age and PSA between the training and external validation cohorts, we performed additional statistical analyses at different age levels (i.e. age < 70 years and age ≥ 70 years) and PSA levels (i.e. 0 ≤ PSA < 10, 10 ≤ PSA < 20, and PSA ≥ 20) to examine the influence of this difference on the classification performance of the DL models (Supplementary Sect. 8). The results of these analyses suggested that the difference in age or PSA between the training and external validation cohorts had little influence on the results of the performance comparison between DL models and PI-RADS assessment (Supplementary Sect. 8 [Table S4]).

#### Comparison of the performance between DL-BM and PI-RADS assessment

**Fig. 3:**
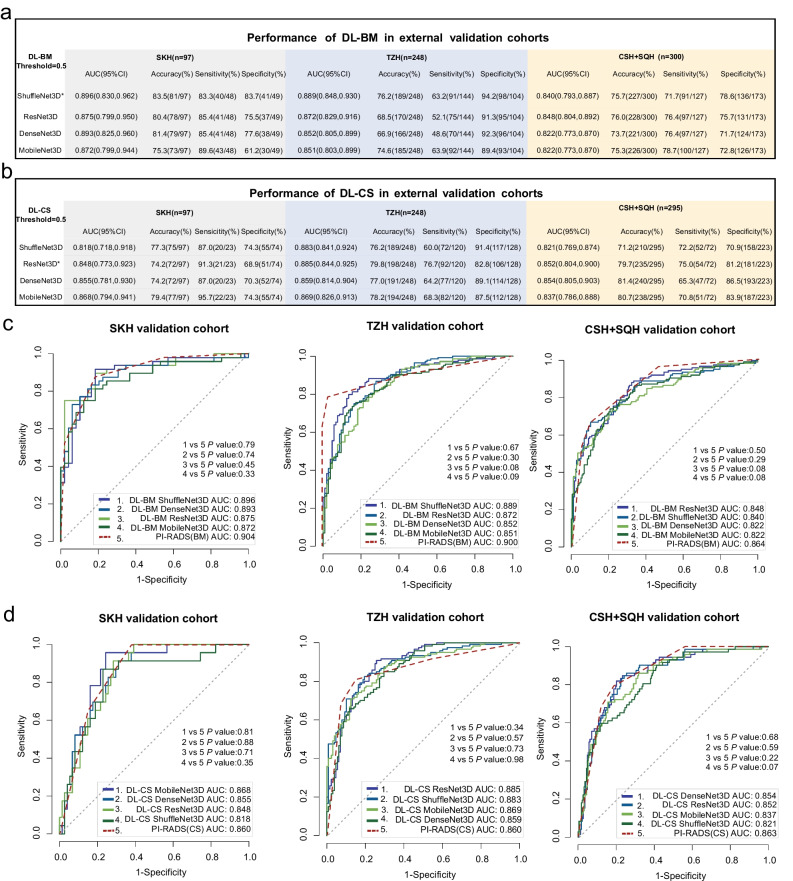
Diagnostic performance of DL-BM and DL-CS models in three external validation cohorts. **a** AUC, sensitivity, and specificity of DL-BM models in each external validation cohort; **b** AUC, sensitivity, and specificity of DL-CS models in each external validation cohort; **c** ROC curves of DL-BM models (solid line) and PI-RADS assessment (dotted line); **d** ROC curves of DL-CS models (solid line) and PI-RADS assessment (dotted line). *The total performance of the model numerically superior to those of the other three models when considering the AUC, sensitivity, and specificity in all external validation cohorts comprehensively. Abbreviation: DL-BM, deep learning models for the classification between benign and malignant lesions; DL-CS, deep learning models for the classification between clinically significant and non-clinically significant prostate cancer; PI-RADS (BM), the assessment with Prostate Imaging and Reporting and Data System (for the classification between benign and malignant lesions); PI-RADS (CS), the assessment with Prostate Imaging and Reporting and Data System (for the classification between clinically significantly and non-clinically significantly prostate cancer); ROC, receiver operating characteristics; AUC, area under ROC curve; SKH, Suzhou Kowloon Hospital; TZH, the People’s Hospital of Taizhou; SQH, the People’s Hospital of Suqian; CSH, Changshu No.1 People’s Hospital; mpMRI, multiparametric MRI

The details of the AUC, accuracy, sensitivity, and specificity of the DL-BM models for the detection of malignant lesions are summarised in Fig. 3a. The accuracy, sensitivity, and specificity were calculated according to the risk probability threshold of 0.5 for each DL-BM model. As shown in Fig. 3a, all DL-BM models (i.e. those based on ShuffleNet3D, ResNet3D, DenseNet3D, and MobileNet3D) achieved excellent performance in the classification between benign and malignant lesions. Figure 3c shows the ROC curves of the DL-BM models based on the above networks (solid line) and PI-RADS assessment (dotted line) for each external validation cohort. As revealed by the Delong tests, the differences in ROC curves between DL-BM models and PI-RADS assessment were not significant in any of the three external validation cohorts (*P* > 0.05) (Fig. 3c). The results suggested that the diagnostic performance of these DL-BM models in the detection of malignant lesions was comparable to that of PI-RADS assessment by expert radiologists.

Although no significant difference (*P* > 0.05, except ShuffleNet3D > MobileNet3D in TZH external validation [*P* = 0.02]) was observed for each paired comparison among the four DL-BM models in each external cohort (Supplementary Sect. 9 [Table S5]), as shown in Fig. 3a, the total performance of the DL-BM model based on the ShuffleNet3D network was numerically superior to those of the other three models when comprehensively considering the AUC, sensitivity, and specificity in all external validation cohorts.

#### Comparison of the performance between DL-CS and PI-RADS assessment

The details of the AUC, accuracy, sensitivity, and specificity of the DL-CS models for the detection of csPCa are summarised in Fig. 3b. The accuracy, sensitivity, and specificity were calculated according to the risk probability threshold of 0.5 for each DL-CS model. As shown in Fig. 3b, all DL-CS models achieved excellent classification performance between csPCa and non-csPCa. Figure 3d shows the ROC curves of the DL-CS models based on the above networks (solid line) and PI-RADS assessment (dotted line) for each external validation cohort. As revealed by the Delong tests, the difference in ROC curves between each DL-CS model and PI-RADS assessment was not significant in any of the three external validation cohorts (*P* > 0.05) (Fig. 3d). The results suggest that the performance of these DL-CS models in the detection of csPCa was comparable to that of the PI-RADS assessment by expert radiologists.

Although no significant difference (*P* > 0.05, except ResNet3D > DenseNet3D in TZH external validation [*P* = 0.04]) was observed for each paired comparison among the four DL-CS models (Supplementary Sect. 9 [Table S6]), as shown in Fig. 3b, the total performance of the DL-CS model based on the ResNet3D network (DL-CS-Res) was numerically superior to those of the other three models when comprehensively considering the AUC, sensitivity, and specificity in all external validation cohorts.

**Fig. 4:**
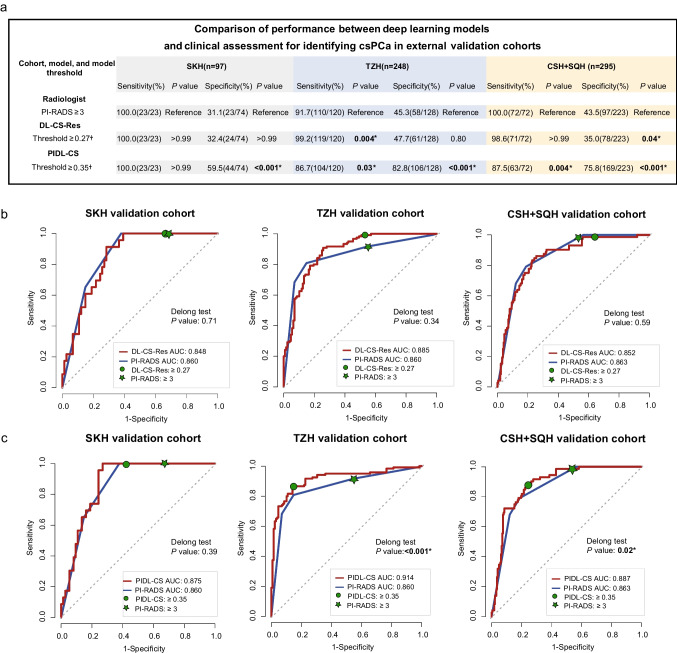
Diagnostic performance of PI-RADS, DL-CS-Res, and PIDL-CS for the detection of csPCa in the external validation cohorts. **a** The sensitivities and specificities of PI-RADS, DL-CS-Res and PIDL-CS in three external validation cohorts at chosen thresholds; **b** threshold points of DL-CS-Res and PI-RADS assessment for the detection of csPCa in three external validation cohorts; **c** threshold points of PIDL-CS and PI-RADS assessment for the detection of csPCa in three external validation cohorts. Receiver operating characteristics curves of DL-CS-Res and PIDL-CS are red lines, and those of PI-RADS assessment are blue lines. Asterisk symbol means significant (*P* < 0.05). Dagger symbol: compared with the PI-RADS assessment with the threshold of ≥ 3. Abbreviation: DL-CS-Res, the deep learning model based on ResNet3D network for the classification between clinically significant and non-clinically significant prostate cancer; PI-RADS, Prostate Imaging and Reporting and Data System; PIDL-CS, integrated model combining DL-CS-Res and PI-RADS assessment; SKH, Suzhou Kowloon Hospital; TZH, the People’s Hospital of Taizhou; SQH, the People’s Hospital of Suqian; CSH, Changshu No.1 People’s Hospital; mpMRI, multiparametric MRI

The performance of DL-CS-Res in the tuning dataset was also numerically superior to those of the other models (Supplementary Sect. 6 [Table S2]). Thus, the sensitivity and specificity of this DL-CS model were compared with those of the PI-RADS assessment. Corresponding to the threshold of PI-RADS score ≥ 3, the risk probability threshold of the DL-CS-Res for the detection of csPCa was set to ≥ 0.27 (Supplementary Sect. 7 Table S3 and Fig. S1[a]). Figure 4a shows the details of sensitivities and specificities of PI-RADS assessment with the threshold of PI-RADS score ≥ 3 and those of DL-CS-Res with a risk probability threshold of ≥ 0.27 for the detection of csPCa in all external validation cohorts. In the SKH cohort, none of the sensitivity and specificity was significant (sensitivity, 100% [23/23] vs. 100% [23/23], *P* > 0.99; specificity, 32.4% [24/74] vs. 31.1% [23/74], *P* > 0.99) between the DL-CS-Res and PI-RADS assessment. In the TZH cohort, compared with PI-RADS assessment, DL-CS-Res showed increased sensitivity (99.2% [119/120] vs. 91.7% [110/120], *P* = 0.004) but comparable specificity (47.7% [61/128] vs. 45.3% [58/128], *P* = 0.80). In the CSH + SQH cohort, compared with PI-RADS assessment, DL-CS-Res showed a comparable sensitivity (98.6% [71/72] vs. 100.0% [72/72], *P* > 0.99) but decreased specificity (35.0% [78/223] vs. 43.5% [97/223], *P* = 0.04). Figure 4b shows the threshold points for the detection of csPCa for PI-RADS and DL-CS-Res in each external validation cohort.

#### Comparison of the performance between PIDL-CS and PI-RADS assessment

**Fig. 5:**
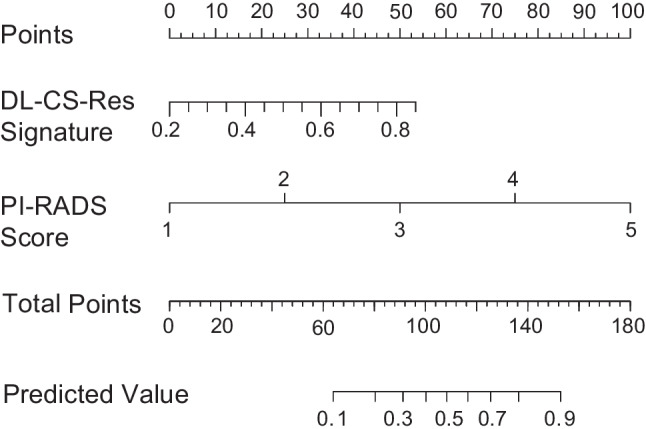
Nomogram of PIDL-CS for the detection of clinically significant prostate cancer. Abbreviation: PI-RADS, Prostate Imaging and Reporting and Data System; DL-CS-Res, the deep learning model based on ResNet3D network for the classification between clinically significant and non-clinically significant prostate cancer; PIDL-CS, integrated model combining DL-CS-Res and PI-RADS assessment

Because DL-CS-Res showed the best performance for the diagnosis of csPCa (Supplementary Sect. 6 [Table S2]) in the tuning dataset, the PIDL-CS was developed by integrating the signature of DL-CS-Res with PI-RADS assessment (Fig. 5). The AUC values of the PIDL-CS for the external validation cohorts of SKH, TZH, and CSH + SQH were 0.875 (95% CI: 0.808, 0.942), 0.914 (95% CI: 0.876, 0.951), and 0.887 (95% CI: 0.848, 0.926), respectively (Fig. 4c). As revealed by the Delong test, the ROC performance of the PIDL-CS model was superior to that of the PI-RADS assessment in the external validation cohorts of TZH (*P* < 0.001, Fig. 4c [middle]) and CSH + SQH (*P* = 0.02, Fig. 4c [right]). However, in the SKH external validation cohort, no significant difference in the ROC performance was observed between the PIDL-CS and PI-RADS assessments (*P* = 0.39, Fig. 4c [left]). Due to the inconsistent results of AUC comparisons in the three external validation cohorts, we performed a comparison of the pooled AUCs of all external validation cohorts between the PIDL-CS and PI-RADS assessment. We found that the pooled AUC of the PIDL-CS from these three cohorts was higher than that of the PI-RADS assessment (Supplementary Sect. 8 [Table S4, Fig. S2]).

Corresponding to the threshold of PI-RADS score ≥ 3, the risk probability threshold of the PIDL-CS for the detection of csPCa was set to ≥ 0.35 (Supplementary Sect. 7 Table S3 and Fig S1[b]). Figure 4a also summarises the details of the sensitivity and specificity of the PIDL-CS for the detection of csPCa with a risk probability threshold of ≥ 0.35 for each external validation cohort. In the SKH cohort, compared with PI-RADS assessment, PIDL-CS showed equal sensitivity (100% [23/23] vs. 100% [23/23], *P* > 0.99) but much higher specificity (59.5% [44/74] vs. 31.1% [23/74], *P* < 0.001). In the TZH cohort and CSH + SQH, compared with PI-RADS assessment, PIDL-CS showed slightly poorer sensitivities (TZH, 86.7% [104/120] vs. 91.7% [110/120], *P* = 0.03; and CSH + SQH, 87.5% [63/72] vs. 100% [72/72], *P* = 0.004) but much higher specificity (TZH, 82.8% [106/128] vs. 45.3% [58/128], *P* < 0.001; and CSH + SQH, 75.8% [169/223] vs. 43.5% [97/223], *P* < 0.001). Figure 4c shows the threshold points for csPCa detection for PI-RADS assessment and PIDL-CS in each external validation cohort. Thus, our proposed PIDL-CS model can greatly reduce the number of unnecessary biopsies compared with PI-RADS assessment.

## Discussion

The present study developed DL models on multicentre bpMRI (T2WI, DWI, and ADC images), which can achieve accurate and robust discrimination between benign and malignant lesions and that between csPCa and non-csPCa. We then compared the performances of these DL models and the integrated model combining the deep learning model and PI-RADS assessment, with that of PI-RADS assessment alone.

## Comparison of the performance between DL-CS and PI-RADS assessment

The present study found that the DL-CS models presented AUCs similar to that of the PI-RADS assessment for the detection of csPCa in each external validation cohort. Furthermore, compared with PI-RADS assessment, the DL-CS-Res showed comparable sensitivity and specificity in each external validation cohort, except for increased sensitivity in the TZH cohort and decreased specificity in the CSH + SQH cohort. Because sensitivity and specificity are dependent on the selection of the risk probability threshold, whereas AUC reflects the overall performance of the classification model, our findings suggest that DL-CS-Res may have a diagnostic performance comparable to that of PI-RADS assessment for the detection of csPCa. Our findings are in agreement with recent studies that reported a similar diagnostic performance of bpMRI and mpMRI [6–8], suggesting that T2WI, DWI, and ADC are closely related to the progression of prostate cancer [2, 5].

Our findings are also consistent with those of Schelb et al. [13] and Netzer et al. [20], who found a comparable diagnostic performance of csPCa between DL models and PI-RADS assessments. As an extension of these previous studies [13, 20], for the comparison with PI-RADS assessment, the present study not only included more samples (*n* = 1861) from seven hospitals in multiple cities but also developed classification models with multicentre training and independent multicentre validation. These multicentre data varied with the differences in scanner manufacturers and scanning parameters. Additionally, in the present study, the multicentre training data (except for the tuning dataset) were augmented to increase diversity further [12]. Such inter-centre diversity in samples effectively reduces the overfitting of the classification model, thereby greatly improving the robustness and generalisation of DL models [21, 22].

The PI-RADS assessment of all MRI images for the diagnosis of csPCa in the present study was performed by five board-certified radiologists with 3–5 years of experience and two expert radiologists with approximately 20 years of experience. Thus, although the DL models of the present study did not outperform the PI-RADS assessment, they presented the potential to not only reduce the inconsistency among radiologists’ PI-RADS assessments but also improve the diagnostic performance of junior radiologists and decrease the workload of expert radiologists.

## Comparison of the performance between PIDL-CS and PI-RADS assessment

In the present study, an increased AUC of PIDL-CS compared to PI-RADS assessment was observed for TZH and CSH + SQH but not for SKH. These findings suggest that in terms of the diagnosis of csPCa, the PIDL-CS presented generally better performance in TZH and CSH + SQH, but a comparable performance in SKH when compared to PI-RADS assessment. A possible explanation for this performance difference is the inconsistencies in image protocols, scanning parameter settings, and even the work state of the MRI scanner among different hospitals. These variations in MRI may have a greater influence on the representation and characterisation of fine image features (e.g. deep textures in lesions) than the evidently visible ones (e.g. the size and intensity of lesions), therefore, to some degree, counteracting the advantages of DL in the characterisation of tumour heterogeneity. Additionally, the number of SKH samples was much smaller than that of TZH and CSH + SQH samples. In fact, for the patients of all three external validation cohorts, the pooled AUC of the PIDL-CS was higher than that of the PI-RADS assessment (Supplementary Sect. 8 [Table S4, Fig. S2]). This may be because TZH and CSH + SQH played dominant roles in the comparison of classification performances.

Although in SKH the AUC of PIDL-CS was comparable to that of the PI-RADS of assessment, the specificity of the former was much higher than that of the latter for the diagnosis of csPCa. Unlike the AUC, which reflects the overall performance of classification models, the specificity and sensitivity indicate the ability of the classification model to discriminate novel samples at a specific risk probability threshold. In addition to SKH, PIDL-CS also presented a much higher specificity for the detection of csPCa than PI-RADS assessment alone in the other external cohorts (i.e. TZH and CSH + SQH). Although in these two cohorts, the sensitivity of PIDL-CS for the detection of csPCa decreased compared to PI-RADS assessment, PIDL-CS presented greater AUCs than those of PI-RADS. This finding suggests that the integration of the DL-CS-Res model and PI-RADS assessment may improve the diagnosis of csPCa and has the potential to aid radiologists in increasing the specificity of the diagnosis of csPCa, thereby reducing unnecessary biopsies.

## Comparison of the performance between DL-BM and PI-RADS assessment

The present study also found that the DL-BM models showed similar performances to the PI-RADS assessment in each external validation cohort for the classification between malignant and benign prostate lesions. Previous studies developed AI models to detect PCa (Yang et al. [32], Wu et al. [33], and Chen et al. [34]). Among them, Chen et al. [34] reported that the performance of the developed model was superior to that of PI-RADS assessment. However, the classification model in the study was based on data from a single centre. As an extension of these studies, we developed DL models based on multicentre data to improve the generalisation and reliability of the models. Thus, DL-BM models have the potential to aid radiologists in finding benign lesions.

Although the NCCN guidelines recommend MRI as the first and most important method for monitoring PCa, some studies have used PET for the diagnosis of PCa (e.g. [35–38]). Among these examples, Yi et al. [35] developed three random forest (RF) models based on PET images from two centres for the classification between PCa and non-PCa. This study found that the performances of these RF models were better than that of the PSA density (PSAD). The AUCs (0.856–0.925) of the RF models in Yi et al. [35] were also slightly higher than those of the DL models in the present study. Although the number of samples in this study (training set: 64, testing set: 36) was much lower than those in the present study (training set: 1216, testing set: 645), this may demonstrate the potential of multimodal images to aid in the precise diagnosis of PCa.

ResNet and ShuffleNet can effectively extract high-dimensional features via residual learning [24, 26]. In the DL framework, the shallow network layers extract simple visual features, such as contours and coarse textures. As the number of network layers increases, a great deal of intrinsic information about lesions that cannot be visually or semantically represented is characterised [24]. Such deep information is very likely to reflect the heterogeneity of the tumour and therefore facilitate the assessment of the aggressiveness of PCa. Thus, in the present study, the classification models with ResNet or ShuffleNet took advantage of the characteristics of the MRI images [15] and then mapped these characteristics into a risk probability (i.e. radiomics signature) as an outcome. Thus, it can provide radiologists with elaborate and quantitative information and therefore aid radiologists, particularly junior radiologists, who are the majority in clinical practice, in the diagnosis of PCa.

## Limitations

The present study has several limitations. The present study was retrospective and used multicentre data. As mentioned in the “Material and Methods” section, to train and test the DL models independently, we divided the data into training and external validation cohorts at the hospital level with no significant difference in the proportion of csPCa between these two cohorts. These classification criteria can reduce the bias of the classification results of DL models to a great degree. However, because the present study was retrospective, it was difficult to achieve a complete balance between the training and external validation cohorts for all characteristics, possibly resulting in some deviations. Therefore, the proposed DL models require further validation using independent prospective data. Second, in the present study, the lesions were delineated manually by expert radiologists. It is considerably time-consuming and laborious to enlarge the dataset. Additionally, inaccurate lesion delineation can adversely affect the model performance. Therefore, it is necessary to develop an accurate and automatic method for lesion segmentation. For example, some networks can be used as segmentation networks (e.g. U-Net and generative adversarial network) to identify lesions and their margins. Then, the ROIs containing the segmented lesions are extracted and used as inputs to the classification models. Additionally, segmentation and classification models can also be carried out in parallel, which is followed by a decision fusion node to achieve computer-aided detection and diagnosis.

In conclusion, this study developed DL models to diagnose csPCa. These models showed a performance comparable to that of PI-RADS assessment. More importantly, when the DL-CS model was integrated with PI-RADS assessment, it greatly increased the specificity of csPCa detection relative to PI-RADS assessment alone. Thus, our DL models can significantly reduce unnecessary biopsies and may aid in the precise diagnosis of csPCa.

## Supplementary Information

Below is the link to the electronic supplementary material.

Supplementary file3 (DOCX 229 KB)

## Data Availability

The related images and clinical data of the present study are not publicly available due to the inclusion of the private information of patients. However, these data can be obtained through institutional approvals as well as the signed agreements of data usage and/or the signed agreements of material transfer.
