# Supplementary material for: Predicting clinically significant prostate cancer with a deep learning approach: a multicentre retrospective study
Source: Eur J Nucl Med Mol Imaging. 2022 Nov 21;50(3):727–41. doi: 10.1007/s00259-022-06036-9 (PMC9852176; doi:10.1007/s00259-022-06036-9)
Supplement: Supplementary file 1 — Supplementary file3 (DOCX 229 KB) [file 259_2022_6036_MOESM1_ESM.docx]

**Supplementary Section**

**Predicting Clinically Significant Prostate Cancer with a Deep Learning Approach: A Multicentre Retrospective Study**

# 1. Magnetic resonance imaging (MRI) images acquisition

**Table S1** Parameters of MRI scanning from seven hospitals

| Hospitals | Sequences | Vendor | MRI strength | B value(sec/mm^2^) | Slice thickness (mm) | Spacing between  slices (mm) | Echo time(s) | Repetition time(s) |
| --- | --- | --- | --- | --- | --- | --- | --- | --- |
| SUH 1^st^ | T2WI, DWI, ADC | Siemens Skyra | 3T | 50/70/1500/2000 | 3 | 3/3.45 | 60/104 | 6540/7590 |
| SUH 2^nd^ | T2WI, DWI, ADC | Philips Ingenia | 3T | 10/20/50/100/200/1000/2000 | 1.5/2/3/3.4/3.5/3.7/  3.8/3.9/4/4.1/4.2/4.3/5 | 1.65/3/3.2/3.3/3.4/3.5/3.7/  3.8/3.9/2.2/4/4.1/4.2/4.3/5 | 77/78/100 | 4542/4828/4898/  4733/4972/ 6000 |
| ZJGH | T2WI, DWI, ADC | Philips achieva | 3T | 0/400/800/1500 | 3 | 3.3 | 62/75 | 2000/4342//6118/  6173/6498/6489 |
| SKH | T2WI, DWI, ADC | Siemens Skyra | 3T | 400/800/1200 | 3.5/4 | 3.5/4 | 72/97 | 3900/4342/6118/  6173/6489/6498/  7500 |
| TZH | T2WI, DWI, ADC | Siemens Skyra and Vero | 3T | 0/50/800/1000/1500 | 3.5/4/5/5.5 | 3.5/4/4.8/6/6.6 | 62/64/74/97/104 | 4480/5000/5100/  7500/8600 |
| SQH | T2WI, DWI, ADC | Philips Ingenia | 3T | 0/500/1000/1400/2000 | 3/4 | 3.5/4.4 | 68/80/110 | 4473/4176/4564/  6600/6700 |
| CSH | T2WI, DWI, ADC | Philips Achieva TX | 3T | 0, 1000, 2000 | 3 | 3 | 76/80 | 2750/3000 |

Abbreviation: *T2WI*, T2-weighted imaging; *DWI*, diffusion-weighted imaging; *ADC*, Apparent diffusion coefficient; *SUH* 1*^st,^* the First Affiliated Hospital of Soochow University; *SUH* 2*^nd^*, the Second Affiliated Hospital of Soochow University; *ZJGH*, the Affiliated Zhangjiagang Hospital of Soochow University; *SKH*, Suzhou Kowloon Hospital; *TZH*, the People's Hospital of Taizhou; *SQH*, the People's Hospital of Suqian; *CSH*, Changshu NO.1 People's Hospital.

# 2. Prostate Imaging Reporting and Data System assessment

All prostate MRI examinations were divided into five groups and performed on the index lesions based on T2-weighted imaging T2WI, diffusion-weighted imaging (DWI)/apparent diffusion coefficient (ADC), and dynamic contrast-enhanced (DCE) images according to the Prostate Imaging and Reporting and Data System version 2.1 [1, 2] respectively, by five board-certified radiologists from SUH 1^st^ and SUH 2^nd^ (reader 1 [J.B.] with 5 years of experience; reader 2 [X.M.Q.] with 3 years of experience; reader 3 [P.F.J.] with 4 years of experience; reader 4 [Y.Y.Z.] with 5 years of experience; and reader 5 [Y.T.J.] with 5 years of experience). They all accessed clinical details including age, prostate-specific antigen (PSA) level, digital rectal examination information, and other risk factors, such as family history and routine habits. Then, all index lesions were re-divided into two groups, for which the results of the PI-RADS assessment of the five radiologists were checked respectively by two expert radiologists from SUH 1^st^ and SUH 2^nd^ (reader 6 [X.M.W.] with 18 years of experience and reader 7 [J.K.S.] with 22 years of experience). The expert radiologists accessed the same clinical details as those accessed by the five radiologists. Any disagreement among the five radiologists and two expert radiologists was discussed until a consensus was reached. All readers were members of the institution’s prostate disease management team with 3-22 years of experience in prostatic MRI and had read more than 1000 prostatic MRI scans using a PI-RADS scoring system at the time of the present study. All readers were blinded to the pathological diagnosis information.

# 3. Histological review

Needle biopsies obtained using transrectal ultrasound-guided systemic biopsy and MRI-guided biopsy guidance were independently reviewed by experienced urological pathologists in the respective hospitals. In this procedure, a “two-step-checking” scheme was used to avoid potential bias, wherein a junior pathologist first read the prostate specimens, and then the reports were checked by a senior pathologist. If there was a disagreement, the reports were discussed until a consensus was reached. Each core containing cancer was assigned to a primary and secondary Gleason grade group. For patients undergoing radical prostatectomy (RP), prostatic specimens were uniformly processed and subjected to histological investigation.

# 4. Data preprocessing

According to the purpose of the present study, image preprocessing was performed on T2WI, high b-value DWI images, and ADC maps derived from the DWI images.

(1) Data de-identification.

The original format of the data in the present study was Digital Imaging and Communications in Medicine (DICOM), which contained patients’ private information such as name and address. The patients’ specific sensitive information was removed by converting data to the Neuroimaging Informatics Technology Initiative (NIFTI) format, abiding by the requirement of data de-identification in the U.S. Health Insurance Portability and Accountability Act (HIPAA) and the E.U. General Data Protection Regulation (GDPR).

(2) Image registration.

In the present study, both DWI and ADC maps were registered to T2WI images for each patient. The aim of the registration was to reduce motion-induced misalignments and remove differences in spatial resolution, such as voxel size and slice thickness, between T2WI and DWI images. Image registration was performed using the SimpleElastix toolbox (http://simpleelastix.github.io/) by applying a translation registration method based on the advanced matte mutual information criterion.

(3) Data augmentation

Data augmentation was performed only for the images of the training cohort, except for the tuning dataset, to enrich data diversity at the training step, thereby alleviating overfitting and improving the generalization of the deep learning (DL) models. In this study, the basic augmentation operations for images comprised translation in a random direction and rotation at a random angle. In the training cohort, the augmented images replaced their original images to train the deep learning models. In contrast, the remaining tuning datasets in the training cohort and external validation cohorts without data augmentation were used for model tuning and testing, respectively.

# 5. Network parameter settings

Each network was trained using cross-entropy loss and the Adam optimizer. The learning rate was set to the initial value of 0.01 and then steadily decreased every five epochs (learning rate new = learning rate old * (0.1 ^epoch/5^). The batch size was set to 32, and the number of epochs was set to 30. We used PyTorch (version 1.12.0; https://pytorch.org) as our code base trained all deep learning (DL) models on a single RTX GPU.

# 6. Development of the integrated model combining DL model and PI-RADS assessment

Table S2 summarizes the area under the receiver operating characteristic curve (AUC), accuracy, sensitivity, and specificity of the DL models for the detection of clinically significant prostate cancer (csPCa) (DL-CS) models in the tuning dataset. The DL-CS model based on ResNet3D (DL-CS-Res) showed higher AUC, accuracy, sensitivity, and specificity values than those of models based on other networks. We then constructed an integrated model (PIDL-CS) combining the deep learning signature of DL-CS-Res and the PI-RADS assessment using logistic regression for the detection of csPCa.

**Table S2** Performance of DL-CS models in the tuning dataset (probability threshold = 0.5)

| DL-CS models | AUC (95%CI) | Accuracy (%) | Sensitivity (%) | Specificity (%) |
| --- | --- | --- | --- | --- |
| ResNet3D | 0.922(0.868,0.975) | 90.2(110/122) | 89.5(34/38) | 90.5(76/84) |
| DenseNet3D | 0.893(0.828,0.957) | 84.4(103/122) | 78.9(30/38) | 86.9(73/84) |
| ShuffleNet3D | 0.856(0.776,0.935) | 83.6(102/122) | 81.6(31/38) | 84.5(71/84) |
| MobileNet3D | 0.922(0.869,0.974) | 89.3(109/122) | 86.8(33/38) | 90.5(76/84) |

Abbreviation: *AUC*, area under receiver operating characteristic curve; *DL-CS*, deep learning models for the classification between clinically significant and non-clinically significant prostate cancer.

# 7. Selection of the probability threshold values of DL-CS-Res and PIDL-CS

In the present study, the PI-RADS threshold for the detection of csPCa was ≥ 3. To compare the specificities of the DL-CS-Res and PIDL-CS with that of PI-RADS assessment, the risk probability thresholds of these classification models were set, such that the absolute value of the difference in the sensitivity between the classification model (i.e., DL-CS-Res or PIDL-CS) and PI-RADS assessment with threshold ≥ 3 was the smallest, whereas the specificity of this classification model was the largest under this sensitivity in the tuning dataset.

As shown in Table S3, in tuning dataset, PI-RADS assessment had a sensitivity of 100% (38/38) and specificity of 44.0% (37/84) for the detection of csPCa with the threshold of PI-RADS score ≥ 3. Corresponding to this threshold, the risk probability threshold of the DL-CS-Res for the detection of csPCa was set as ≥ 0.27, with which the DL-CS-Res showed the same sensitivity as that of PI-RADS assessment 100.0% (38/38), and at the same time had the largest specificity 50.0% (42/84) in the tuning dataset. Fig S1(a) showed the threshold points of the detection of csPCa for PI-RADS and DL-CS-Res in the tuning dataset, respectively. This probability threshold was used to evaluate the performances of DL-CS-Res in each external validation cohort.

As shown in Table S3, in tuning dataset, PIDL-CS showed the same sensitivity as that of PI-RADS assessment 100.0% (38/38), and at the same time had the largest specificity 76.2% (64/84) in the tuning dataset. Fig S1(b) showed the threshold points of the detection of csPCa for PI-RADS and PIDL-CS in the tuning dataset, respectively. This probability threshold was used to evaluate the performances of PIDL-CS in each external validation cohort.

**Table S3 Sensitivity and specificity of DL-CS-Res, PIDL-CS and PI-RADS assessment for the detection of csPCa in the tuning dataset**

| Model and model threshold | Sensitivity (%) | *P* value | Specificity (%) | *P* value |
| --- | --- | --- | --- | --- |
| **Radiologists**  PI-RADS ≥ 3 | 100.0(38/38) | Reference | 44.0(37/84) | Reference |
| **DL-CS-Res**  Threshold ≥ 0.27† | 100.0(38/38) | > 0.99 | 50.0(42/84) | 0.49 |
| **PIDL-CS**  Threshold ≥ 0.35† | 100.0(38/38) | > 0.99 | 76.2(64/84) | **< 0.001*** |

Note: * Significant (*P* < 0.05)

† Compared with PI-RADS assessment with threshold of ≥ 3

Abbreviation: *DL-CS-Res*, the deep learning model based on ResNet3D network for the classification between clinically significant and non-clinically significant prostate cancer; *PI-RADS*, Prostate Imaging and Reporting and Data System; *PIDL-CS*, integrated model combining DL-CS-Res and PI-RADS assessment.


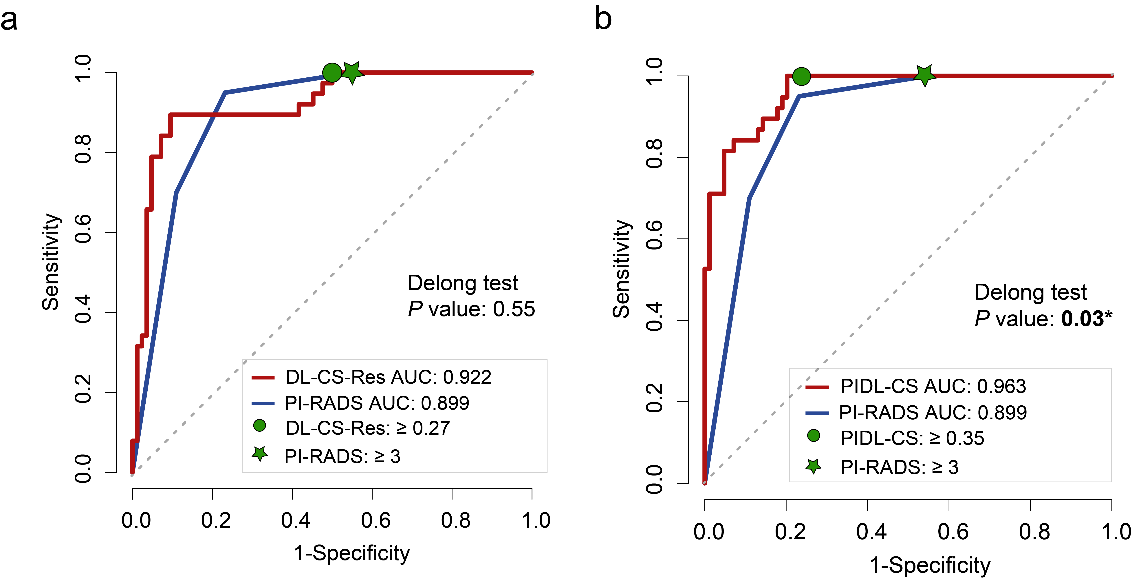


**Fig. S1** Threshold points of DL-CS-Res, PIDL-CS and PI-RADS assessment for the detection of csPCa in the tuning dataset. (**a)** The threshold points of DL-CS-Res (green circle) and PI-RADS assessment (green star) for the detection of csPCa in the tuning dataset; (**b)** The threshold points of PIDL-CS (green circle) and PI-RADS assessment (green star) for the detection of csPCa in the tuning dataset. Receiver operating characteristics curves of DL-CS-Res and PIDL-CS are red lines, and those of PI-RADS assessment are blue lines.

* Significant (*P* < 0.05)

Abbreviation: *DL-CS-Res*, the deep learning model based on ResNet3D network for the classification between clinically significant and non-clinically significant prostate cancer; *PI-RADS*, Prostate Imaging and Reporting and Data System; *PIDL-CS*, integrated model combining DL-CS-Res and PI-RADS assessment.

# 8. Subgroup analysis for different age and PSA (prostate-specific antigen) levels

In the present study, age and PSA levels of patients between the training and external validation cohorts were significantly different. To address this problem, we performed an additional analysis. Specifically, we divided the total patients of all three external validation cohorts into two groups according to their age (i.e., age < 70 years and age ≥ 70 years) and into three groups according to their PSA level (i.e., 0 ≤ PSA < 10, 10 ≤ PSA < 20, and PSA ≥ 20). The present study focused on comparing the diagnostic performance of csPCa between deep learning models and PI-RADS assessments. Therefore, for each age level and PSA level, we compared the AUCs of the deep learning model DL-CS-Res and the integrated model PIDL-CS with that of PI-RADS assessment, respectively. We also conducted performance comparisons for the patients at all levels. The comparison results are summarized in Table S4.

As indicated by Table S4, for each age level and PSA level, DL-CS-Res presented an AUC comparable to that of the PI-RADS assessment (*P* > 0.05). These results were consistent with the total results for all the combined levels (DL-CS-Res, AUC = 0.851[0.821, 0.877] vs. PI-RADS: AUC = 0.850[0.820, 0.877], *P* = 0.99) (Fig. S2[a]). For PIDL-CS, for each age level and PSA level, it presented a higher AUC than that of the PI-RADS assessment (*P* < 0.05), except for the level of PSA ≥ 20, at which a marginally higher AUC (*P* = 0.064) was observed. These results were consistent with those for all combined levels (PIDL-CS: AUC = 0.881[0.853,0.905] vs PI-RADS: AUC = 0.850[0.820,0.877], *P* < 0.001) (Fig. S2[b]). Additionally, as shown in Fig 4, DL-CS-Res presented an AUC comparable to that of PI-RADS assessment in each external cohort (*P* > 0.05). PIDL-CS presented a higher AUC for the People's Hospital of Taizhou (TZH), and Changshu NO.1 People's Hospital (CSH) + the People's Hospital of Suqian (SQH) (*P* < 0.05) but a comparable AUC for Suzhou Kowloon Hospital (SKH) when compared to PI-RADS assessment (*P* > 0.05). It should be noted that the samples of SKH (i.e., 97 patients) were much smaller than those of TZH (248 patients) and CSH + SQH (295 patients). Therefore, the performance of PIDL-CS was better than that of PI-RADS assessment. Thus, for both the DL-CS-Res model and PIDL-CS, the overall performances of the diagnosis of csPCa for each age level and PSA level were consistent with that of all combined levels, suggesting that the difference in age or PAS between the training and external validation cohorts had little influence on the results of the performance comparison between deep learning models and PI-RADS assessment.

**Table S4** Subgroup analysis for the comparisons of the diagnostic performance between DL-CS-Res or PIDL-CS and PI-RADS assessment for all patients of three external validation cohorts

| Patient group | PI-RADS  AUC (95%CI) | DL-CS-Res  AUC (95%CI) | *P* value* | PIDL-CS  AUC(95%CI) | *P* value # |
| --- | --- | --- | --- | --- | --- |
| Patients of all levels | 0.850(0.820,0.877) | 0.851(0.821,0.877) | 0.99 | 0.881(0.853,0.905) | <0.001 |
| **Age** |  |  |  |  |  |
| Age < 70 | 0.881(0.837,0.916) | 0.898(0.856,0.931) | 0.43 | 0.918(0.879,0.947) | 0.001 |
| Age ≥ 70 | 0.825(0.781,0.862) | 0.808(0.764,0.848) | 0.43 | 0.849(0.808,0.884) | 0.017 |
| **PSA** |  |  |  |  |  |
| 0≤PSA<10 | 0.789(0.743,0.846) | 0.859(0.810,0.900) | 0.29 | 0.861(0.811,0.901) | 0.049 |
| 10≤PSA<20 | 0.839(0.765,0.897) | 0.845(0.772,0.902) | 0.89 | 0.881(0.813,0.930) | 0.046 |
| PSA≥20 | 0.723(0.664,0.777) | 0.741(0.683,0.793) | 0.58 | 0.759(0.702,0.810) | 0.064 |

Note: *P* value were calculated by Delong test

* Comparison of the AUC of DL-CS-Res with that of PI-RADS assessment.

# Comparison of the AUC of PIDL-CS with that of PI-RADS assessment.

Abbreviation: *DL-CS-Res*, the deep learning model based on ResNet3D network for the classification between clinically significant and non-clinically significant prostate cancer; *PI-RADS*, Prostate Imaging and Reporting and Data System; *PIDL-CS*, integrated model combining DL-CS-Res and PI-RADS assessment; *AUC*, area under receiver operating characteristic curve; *PSA*, Prostate specific antigen.


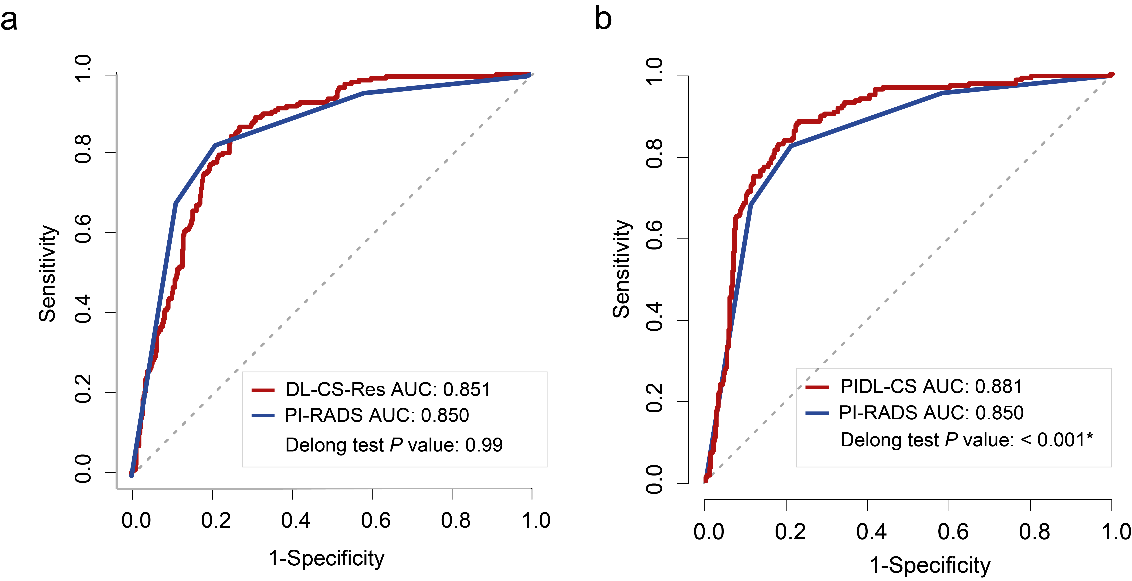


**Fig. S2** ROC curves of DL-CS-Res, PIDL-CS and PI-RADS assessment for the detection of csPCa for all patients of all external validation cohorts. (**a)** ROC curves of DL-CS-Res (Red line) and PI-RADS assessment (Blue line) for the detection of csPCa for all patients of all external validation cohorts; (**b)** ROC curves of PIDL-CS (Red line) and PI-RADS assessment (Blue line) for the detection of csPCa for all patients of all external validation cohorts.

* Significant (*P* < 0.05)

Abbreviation: *DL-CS-Res*, the deep learning model based on ResNet3D network for the classification between clinically significant and non-clinically significant prostate cancer; *PI-RADS*, Prostate Imaging and Reporting and Data System; *PIDL-CS*, integrated model combining DL-CS-Res and PI-RADS assessment; *ROC*, Receiver operating characteristics curves.

# 9. Comparison of performance between deep learning models in external validation cohorts

Results of the paired comparisons of the receiver operating characteristic curves among the DL-BM models and those among the DL-CS models in each external validation cohort are summarised in Table S5 and Table S6, respectively. No significant difference (*P* > 0.05, except ShuffleNet3D > MobileNet3D in TZH external validation [*P* = 0.02]) was observed for each paired comparison among the DL-BM models for malignancy detection (Table S5). No significant difference (*Ps* > 0.05, except ResNet3D > DenseNet3D in TZH external validation [*P* = 0.04]) was observed for each paired comparison among the DL-CS models for csPCa detection (Table S6).

**Table S5** Paired comparisons of the ROC curves among the DL-BM models in each external validation cohort

| The pairs of comparison |  | *P* value | | |
| --- | --- | --- | --- | --- |
|  |  | SKH | TZH | CSH+SQH |
| ShuffleNet3D vs ResNet3D |  | 0.41 | 0.47 | 0.64 |
| ShuffleNet3D vs DenseNet3D |  | 0.88 | 0.06 | 0.27 |
| ShuffleNet3D vs MobileNet3D |  | 0.35 | **0.02*** | 0.24 |
| ResNet3D vs DenseNet3D |  | 0.23 | 0.30 | 0.06 |
| ResNet3D vs MobileNet3D |  | 0.93 | 0.37 | 0.16 |
| DenseNet3D vs MobileNet3D |  | 0.43 | 0.98 | > 0.99 |

Note: *P* value were calculated by Delong test

* Significant (*P* < 0.05)

Abbreviation: *DL-BM*, deep learning models for the classification between benign and malignant lesions; *SKH*, Suzhou Kowloon Hospital; *TZH*, the People's Hospital of Taizhou; *SQH*, the People's Hospital of Suqian; *CSH*, Changshu NO.1 People's Hospital; *ROC*, receiver operating characteristics.

**Table S6** Paired comparisons of the ROC curves among the DL-CS models in each external validation cohort

| The pairs of comparison |  | *P* value | | |
| --- | --- | --- | --- | --- |
|  |  | SKH | TZH | CSH+SQH |
| ResNet3D vs DenseNet3D |  | 0.70 | **0.04*** | 0.86 |
| ResNet3D vs ShuffleNet3D |  | 0.45 | 0.89 | 0.13 |
| ResNet3D vs MobileNet3D |  | 0.50 | 0.38 | 0.20 |
| DenseNet3D vs ShuffleNet3D |  | 0.26 | 0.14 | 0.10 |
| DenseNet3D vs MobileNet3D |  | 0.64 | 0.56 | 0.17 |
| ShuffleNet3D vs MobileNet3D |  | 0.15 | 0.46 | 0.43 |

Note: *P* value were calculated by Delong test

* Significant (*P* < 0.05)

Abbreviation: *DL-CS*, deep learning models for the classification between clinically significant and non-clinically significant prostate cancer; *SKH*, Suzhou Kowloon Hospital; *TZH*, the People's Hospital of Taizhou; *SQH*, the People's Hospital of Suqian; *CSH*, Changshu NO.1 People's Hospital; *ROC*, receiver operating characteristics.

# References

1. Weinreb JC, Barentsz JO, Choyke PL, Cornud F, Haider MA, Macura KJ, et al. PI-RADS Prostate Imaging - Reporting and Data System: 2015, Version 2. Eur Urol. 2016;69:16-40. <https://doi.org/10.1016/j.eururo.2015.08.052>.

2. Turkbey B, Rosenkrantz AB, Haider MA, Padhani AR, Villeirs G, Macura KJ, et al. Prostate Imaging Reporting and Data System Version 2.1: 2019 Update of Prostate Imaging Reporting and Data System Version 2. Eur Urol. 2019;76:340-51. <https://doi.org/10.1016/j.eururo.2019.02.033>.
